# Spiritualität und Behandlung. Die Perspektive von Patienten mit chronischen Schmerzen

**DOI:** 10.1007/s00482-020-00524-3

**Published:** 2021-01-08

**Authors:** Horst Rettke, Rahel Naef, Michael Rufer, Simon Peng-Keller

**Affiliations:** 1 Zentrum für Klinische Pflegewissenschaft, UniversitätsSpital Zürich, Rämistrasse 100 (SHM 26 B6), 8091 Zürich, Schweiz; 2 Klinik für Psychiatrie, Psychotherapie und Psychosomatik, Psychiatrische Universitätsklinik Zürich, Zürich, Schweiz; 3 Professur für Spiritual Care, Theologische Fakultät, Universität Zürich, Zürich, Schweiz

**Keywords:** Multimodale Schmerztherapie, Spirituelle Ressourcen, Chronische Erkrankung, Fokusgruppen, Thematische Analyse, Multimodal pain therapy, Spiritual Resources, Chronical illness, Focus groups, Thematic analysis

## Abstract

**Hintergrund:**

Chronischer Schmerz betrifft alle menschlichen Lebensdimensionen und wirft auch spirituelle Fragen auf, die im Rahmen eines multimodalen Behandlungsmodells berücksichtigt werden sollten.

**Ziel der Arbeit:**

Wir untersuchten die Perspektive von Patienten mit chronischen Schmerzen zu spirituellen Themen und deren möglicher Integration in den Behandlungsprozess.

**Material und Methoden:**

Es wurden fünf Fokusgruppen- und zwei Kleingruppeninterviews an fünf Studienzentren durchgeführt. Daran nahmen 42 Patienten mit chronischen Schmerzen teil, die aktuell in ambulanter oder stationärer Schmerzbehandlung waren. Die Interviews wurden transkribiert und einer thematischen Analyse unterzogen.

**Ergebnisse:**

Drei Themen traten hervor: 1. Chronischer Schmerz durchdringt die gesamte menschliche Existenz. 2. Spirituelle Ressourcen stellen eine Möglichkeit im Umgang mit chronischen Schmerzen dar. 3. Patienten ist es ein Anliegen, mit Fachpersonen in einen Dialog treten zu können, der auch für spirituelle Themen offen ist. Diese haben aus Sicht der Teilnehmenden große Relevanz. Sie verknüpften sie vielfach, aber nicht ausschließlich mit religiösen Überzeugungen. Häufig wurde geschildert, in der Schmerzerfahrung nicht ernst genommen zu werden.

**Diskussion:**

Strategien für einen effektiven Umgang mit chronischem Schmerz zu finden, stellt einen Wendepunkt im Leben dar. In diesem Prozess unterstützt ein offener Dialog mit Fachpersonen, der auch spirituellen Themen Rechnung trägt.

## Abstract (en)

**Background:**

Chronic pain affects all aspects of human life, which raises spiritual questions that should be included within the framework of multimodal care.

**Objectives:**

We investigated the perspective of patients with chronic pain around spiritual concerns and their potential integration into care.

**Materials and methods:**

We conducted five focus group interviews and two small group interviews. In total, 42 patients with chronic pain in outpatient or inpatient pain care at the time of the study participated. The interviews were transcribed and thematically analyzed.

**Results:**

Three themes emerged: (1) Chronic pain permeates the entity of a person’s existence. (2) Spiritual resources are potentially supportive in living with chronic pain. (3) Patients appreciate the opportunity to engage with health care professionals in a dialog that encompasses spiritual concerns. For participants, these concerns have considerable relevance. In many cases participants associated them with religious convictions, but not exclusively. They often related feeling that their pain experience was dismissed.

**Conclusion:**

Finding strategies for effectively dealing with chronic pain represents a turning point in life. Open discussion with health care professionals that allow for spiritual issues facilitates this process.

## Hintergrund und Fragestellung

Chronischer Schmerz zeigt sich aus der Patientenperspektive als schwer zu bewältigende Erfahrung, die alle menschlichen Dimensionen umfasst. Dem versucht eine multimodale Schmerztherapie im Rahmen des biopsychosozialen Ansatzes zu entsprechen. Die Weltgesunheitsorganisation (WHO) ergänzte diesen Behandlungsansatz bereits 1984 um die spirituelle Dimension [25]. Aus Patientensicht spielt diese eine gleichberechtigte Rolle in der Schmerzerfahrung und sollte deshalb im Behandlungsprozess berücksichtigt werden.

Ungeachtet seiner großen Verbreitung [5] wird chronischer Schmerz im klinischen Kontext häufig nicht ausreichend beachtet und trotz erheblicher Fortschritte in der Schmerzmedizin nicht zufriedenstellend behandelt [19, 34]. Chronischer Schmerz belastet die Betroffenen in allen Lebensbereichen stark [19] und kann zur lebensbestimmenden Komponente werden, die unablässig alles Denken und Handeln beherrscht [21]. Bendelow [3] vergleicht diesen Zustand mit dem in der Hospizbewegung gründenden Begriff des „total pain“; einem Schmerzerleben, das in seiner Totalität alle Aspekte menschlichen Daseins umschließt, auch den spirituellen. Chronischer Schmerz stellt ein komplexes Phänomen dar und ein Behandlungsansatz muss dieser Komplexität entsprechen [8]. Genau hier schließt das biopsychosoziale Modell an, das Patienten in ihrer Gesamtheit und das Krankheitserleben im Kontext der individuellen Umstände berücksichtigt [11]. Dieses Modell ergänzte die WHO bereits 1984 um die spirituelle Dimension, die sie gleichbedeutend neben die körperliche, psychische und soziale Dimension stellt und mit ihnen verknüpft [35].

Wiederkehrend wird berichtet, dass behandelnde Fachpersonen bei Patienten mit chronischen Schmerzen am Vorhandensein des Leidens in seinem subjektiv erlebten Ausmaß zweifeln [6, 33] oder den Patienten sogar mit Desinteresse oder Unwillen begegnen, was den Leidensdruck noch erhöht [13]. Ohne plausible Erklärung für das Vorhandensein von chronischem Schmerz scheint die Berechtigung zu fehlen, ihn zu erleiden [14]. Dies treibt manche Patienten dazu an, unaufhörlich nach einer Legitimation ihrer Schmerzsymptomatik [6] und nach vollständiger Heilung zu suchen, die es im biomedizinischen Sinn nicht gibt [33].

Auf dem unumgänglichen Weg, mit chronischem Schmerz leben zu lernen [6, 13, 29, 32], brauchen Betroffene einen partnerschaftlichen Behandlungsansatz mit Fachpersonen, die sie in ihrer Gesamtheit wahrnehmen, nicht an der Schwere ihres Leidens zweifeln und zur Begleitperson werden. Ein um die spirituelle Dimension ergänzter Behandlungsansatz schließt damit Fragen und Suchen nach Identität, Sinn, Bedeutung und Zweck ein [31]. Spirituelle Themen spielen im Umgang mit chronischen Schmerzen bei manchen Patienten eine ausschlaggebende Rolle [9] und stehen in Zusammenhang mit Schmerztoleranz, Stimmung und Lebenszufriedenheit [31].

Die Bedeutung von Spiritualität für diese Patientengruppe wurde bereits mehrfach untersucht und bestätigt [7, 28]. Jedoch ist noch wenig bekannt, wie die Integration spiritueller Themen in eine multimodale Behandlung von Patienten mit chronischen Schmerzen gestaltet werden könnte. Deshalb befragten wir im Rahmen eines größeren Forschungsprojekts sowohl Patienten als auch Fachpersonen [23]. In dieser Publikation berichten wir ausschließlich über die Interviews mit Patienten. Ziel der vorliegenden Arbeit ist es, die Perspektive von Patienten mit chronischen Schmerzen in Hinblick auf die uns leitende Frage zu untersuchen, ob und in welcher Weise es von Patienten gewünscht wird, dass spirituelle Aspekte im Rahmen der Behandlung zur Sprache kommen. Ausgehend von der aktuellen Forschungsdiskussion [24, 25] und Konsensdefinitionen [17, 26] gehen wir von einem Verständnis von Spiritualität aus, das religiöse und nichtreligiöse Überzeugungen, Einstellungen, Erfahrungen und Praktiken umfasst, die a) mit einer Sinnorientierung besonderer Qualität verknüpft sind, b) die Verbundenheit mit dem, was im Leben trägt, inspiriert und integriert, zum Ausdruck bringen und c) das eigene Leben in einem Sinnzusammenhang umfassender Art verorten.

## Studiendesign und Untersuchungsmethoden

Im Rahmen eines qualitativ-explorativen Studiendesigns befragten wir Patienten, die wegen chronischer Schmerzen in ambulanter oder stationärer Behandlung in der Deutschschweiz waren. Wir fokussierten auf Patienten in Einrichtungen, die auf Schmerzbehandlung spezialisiert sind, wie z. B. Fachkliniken oder Schmerzambulatorien. Der qualitative Ansatz bot sich an, um die Erfahrungen im Kontext ihres Erscheinens explorieren und deren Bedeutung aus Sicht der Teilnehmenden heraus verstehen zu können [10].

### Setting und Teilnehmende

Um der Bandbreite der Behandlungssettings gerecht zu werden, wurden unterschiedliche Einrichtungen gezielt angefragt. Zu den fünf teilnehmenden Studienzentren gehörten eine Rehabilitationseinrichtung mit psychosomatischer Spezialisierung, eine Fachklinik mit christlich-religiösem Hintergrund, eine Facharztpraxis für Rheumatologie und Schmerzbehandlung, ein Schmerzambulatorium an einem Universitätsspital und eine Spezialklinik für Akutbehandlung und Rehabilitation. Patienten waren zur Studienteilnahme aufgrund folgender Kriterien geeignet: Alter ≥ 18 Jahre, persistierende Schmerzen seit ≥ 6 Monaten, bestätigte medizinische oder Pflegediagnose „chronischer Schmerz“ und eine Schmerzintensität ≥ 5 während der letzten Schmerzepisode, gemessen auf einer 10-Punkte-Schmerzskala (0 = kein Schmerz, 10 = stärkster vorstellbarer Schmerz; [19]). Die Deutschkenntnisse mussten genügen, um die eigene Situation differenziert verbalisieren und dem Interviewverlauf folgen zu können. Ausgeschlossen wurden Patienten mit akut bedrohlicher Erkrankung wie einer Krebserkrankung, weil damit eine spirituelle Thematik eher im Zusammenhang mit Lebensende und Sterben im Vordergrund steht. Ebenfalls ausgeschlossen wurden Patienten mit kognitiver Beeinträchtigung.

### Rekrutierung

**Figure 1:**
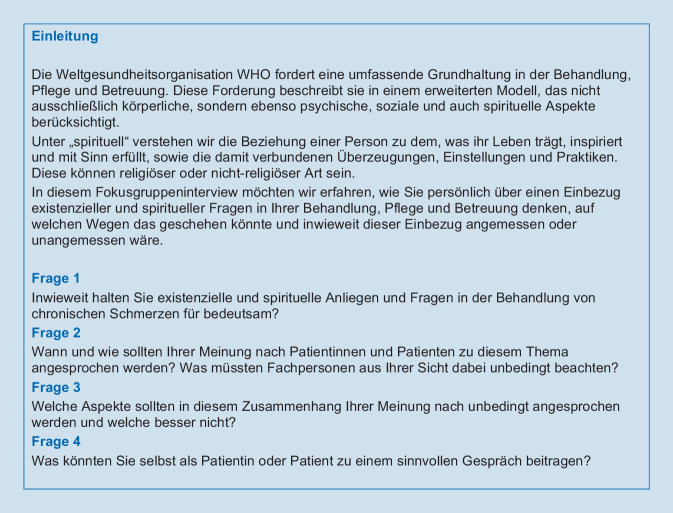

Die ärztliche Leitung jedes Studienzentrums oder eine von ihr beauftragte Person (z. B. Assistentin oder Study Nurse) fragte Patienten zur Teilnahme am Fokusgruppeninterview zu einem im voraus festgelegten Zeitpunkt an. Sie händigte bei Interesse die Studieninformation aus, holte bei definitiver Zusage das schriftliche Einverständnis ein und überreichte den Interviewleitfaden zur individuellen Vorbereitung (Abb. 1) zusammen mit einem anonymisierten soziodemografischen Fragebogen.

### Datensammlung

Wir führten Fokusgruppeninterviews (FGI) durch, weil sie eine Dynamik anstoßen, in der Teilnehmende aufeinander Bezug nehmen [16] und so Interviewthemen vertieft behandeln können. Ebenfalls können so kulturelle Werte zur Sprache gebracht werden. Pelz et al. [22] betonen zudem, dass diese Methode einen Überblick über die Variationsbreite und Struktur von Meinungen und Einstellungen verschafft. Die FGI moderierten ein Pflegewissenschaftler und eine Theologin anhand des Leitfadens gemeinsam. Beide brachten klinische Erfahrung und Expertise in Interviewtechniken ein. Direkt nach jedem FGI tauschten sie ihre Eindrücke zu Inhalt und Dynamik aus. Alle FGI wurden digital aufgezeichnet und von einer Drittperson wörtlich transkribiert und dabei anonymisiert.

### Datenanalyse

**Figure 2:**
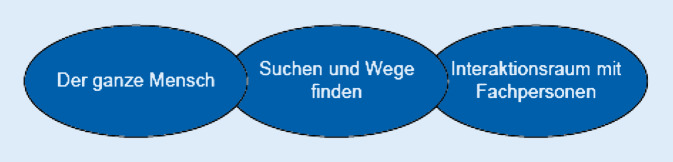

Die Interviewdaten wurden mit der Methode der thematischen Analyse aufgeschlüsselt. Mit ihr lassen sich Muster in qualitativen Daten identifizieren, analysieren und zur Darstellung bringen [4]. Wir entschieden uns innerhalb dieser Methode, den gesamten Datensatz induktiv zu analysieren, um auch latent vorhandene Themen aufzuspüren [4]. Dazu wurden die Transkripte wiederholt gelesen, die einzelnen Interviewbeiträge thematisch codiert und die Codes wiederum zu Themen gebündelt. Diese wurden schrittweise in drei Kernthemen integriert. Dadurch konnten die Ergebnisse auf abstrakter Ebene zusammenfassend benannt und beschrieben, aber dennoch in ihrer Bandbreite dargestellt werden (Abb. 2). Zur Überprüfung der Vollständigkeit und Schlüssigkeit wurden anschließend alle Transkripte in toto gelesen. Die thematische Analyse führte der Pflegewissenschaftler durch und diskutierte die Ergebnisse in regelmäßigen Abständen mit einer weiteren Pflegewissenschaftlerin und einem Theologen. Sie waren nicht an der Interviewführung beteiligt, wohl aber mit Teilen der Transkripte vertraut. Die Diskussionen dienten dem kritischen Prüfen der interpretativen Entscheidungen und dem Untermauern der Vertrauenswürdigkeit der Resultate [30]. Auszüge aus den Interviewbeiträgen unterstützen als Ankerbeispiele wiederum die Bestätigbarkeit der Daten [18].

## Ergebnisse

Insgesamt beteiligten sich 42 Patienten in fünf Studienzentren. In zwei Studienzentren erschienen zum vereinbarten Termin nur drei respektive zwei Teilnehmende. Um auch deren Meinungen und Erfahrungen berücksichtigen zu können, wurden sie in Kleingruppen interviewt. In einem neuen Anlauf gelang auch dort das Rekrutieren einer vollzähligen Fokusgruppe. Die Charakteristika der Teilnehmenden sind in Tab. 1 zusammengefasst.

**Table 1:** 

		*n*	%	MW	SD	Ra
Anzahl Teilnehmende	Fokusgruppe	**–**	**–**	7	–	4–11
	Kleingruppe	–	–	2,5	–	2–3
Geschlecht weiblich	–	28	67	–	–	–
Alter in Jahren	–	–	–	51,20	±13,65	22–80
Schmerzstärke in den letzten 2 Wochen	–	–	–	6,59	±1,66	3–10
Glaubensgemeinschaft	Röm.-kath.	10	24	–	–	–
	Evang.-ref.	15	36	–	–	–
	Andere christl.	7	16	–	–	–
	Islamisch	3	7	–	–	–
	Konfessionslos	7	16	–	–	–

### Kernthemen in der thematischen Darstellung

Alle Interviewbeiträge lassen sich zu folgenden Kernthemen bündeln (Abb. 2):

1. „Der ganze Mensch“ – chronischer Schmerz durchdringt die gesamte menschliche Existenz: Hier nehmen Teilnehmende Bezug auf ihr Menschsein, wenn sie ihr Leben mit den Symptomen und ihre Erfahrungen im Behandlungsprozess beschreiben.
2. „Suchen und Wege finden“ – spirituelle Ressourcen im Leben mit chronischen Schmerzen: Teilnehmende evaluieren hier bewährte oder neue Strategien im Umgang mit chronischem Schmerz. Dies schließt spirituelle Aspekte mit ein.
3. „Ein Interaktionsraum mit Fachpersonen“ – Hinweise zum Einbezug der spirituellen Dimension in die Behandlung: Die Teilnehmenden benennen hier das Anliegen, in einen Dialog mit Fachpersonen treten zu können, der geprägt ist von Wertschätzung und der, wo angezeigt, spirituelle Anliegen und Bedürfnisse unbedingt berücksichtigt.

### „Der ganze Mensch“ – chronischer Schmerz durchdringt die gesamte menschliche Existenz

Betroffene schildern, dass sie sich im Behandlungsprozess häufig auf den chronischen Schmerz reduziert sehen. *„Es geht immer um das Ganze, und so lange man den Patienten nur von seiner Krankheit her anfasst, fehlt immer irgendetwas“* (3, 00:03:14).^1^ Dies widerspricht ihrem Selbstverständnis als Mensch mit chronischen Schmerzen: *„Geist, Seele und Körper gehören zusammen. […] Das kann man gar nicht trennen. Und ich meine, wenn der Körper leidet, dann darf man den Geist nicht vergessen. Und wenn man die Seele stärken kann mit Psychotherapie und Körper mit Physiotherapie, sollte man auch den Geist stärken“* (5, 00:58:42). *„Der Mensch ist ja immer ein Ganzes“* (3, 00:21:03), bringt es eine Teilnehmende auf den Punkt, und ein anderer ergänzt: *„Die Schmerzen betreffen ja jeden Lebensbereich“* (3, 00:25:13). Dies schließt auch die spirituelle Dimension nicht aus. *„Jeder Mensch hat ein Fundament. Irgendein Fundament. Und das kann natürlich erschüttert sein“* (2. 00:04:06).

Weil nicht sichtbar, bleibt die Schmerzerfahrung nur schwer mitteilbar und für Drittpersonen ebenso schwer zugänglich. *„Ich habe starke Schmerzen, aber niemand sieht es und das ist schwierig, auch für die anderen Leute zu verstehen“* (4.1, 00:33:57). Chronischer Schmerz vermag die Grundfeste der Existenz zu erschüttern. *„Die Akzeptanz, dass mir niemand helfen kann, macht mir am meisten weh. Seelisch. Weil ich weiß, ich bin eigentlich auf mich (zurückgeworfen)“* (1, 00:41:15). Die Betroffenen müssen sich langfristig auf ein Leben mit den Symptomen einstellen und manche gehen davon aus, dass die Symptome bestehen bleiben. *„Wir finden keine Heilung. Wir werden mehrere Tabletten am Tag schlucken, verschiedene Therapien und es hilft nichts“* (1, 01:05:12). Zur Schmerzerfahrung gesellen sich wirtschaftliche Sorgen und soziale Einschränkungen. *„Ich habe wegen den Schmerzen meinen Job verloren. Meine Situation wird nicht besser. Und dann sehe ich nur schwarz“* (5, 00:12:47). Eine andere Betroffene führt aus: *„Du entfernst dich von allen Kolleginnen, der Familie. Du sperrst dich eigentlich ein und willst deinen Schmerz einfach weghaben und dadurch verlierst du eigentlich alles“* (1, 00:09:54).

Damit nimmt chronischer Schmerz eine zentrale Rolle im Leben der Teilnehmenden ein. Unweigerlich stellen sich existenzielle Fragen nach Bedeutung und Sinn des Schmerzerlebens. *„Die Schmerzen sind ja chronisch da. Die begleiten jetzt mein Leben. Und da kommt einfach schon die Frage: Was haben die Schmerzen, die eigentlich für mich in dem Sinn keinen Zweck haben, aber welche Bedeutung steht dahinter?“* (5, 01:03:26). In ihrem Leiden, das sie in ihrer gesamten Existenz betrifft, fühlen sich viele Teilnehmende von Fachpersonen unverstanden, nicht ernst genommen und in ihrem Leiden verkannt. *„Was für mich immer das Schlimmste gewesen ist, wenn man das Gefühl gehabt hat, was man erzählt, wird heruntergespielt“* (3, 00:27:44). Diese Art Erfahrungen kann verletzen. Positive Erfahrungen bewirken das Gegenteil. *„Man fühlt sich manchmal nicht ernst genommen. Aber wenn man sich ernst genommen fühlt, hilft das schon viel“* (5, 00:43:11).

### „Suchen und Wege finden“ – spirituelle Ressourcen im Leben mit chronischen Schmerzen

Für Teilnehmende ist es eine wiederkehrende Herausforderung, dem chronischen Schmerz einen angemessenen Platz im Leben zuzuweisen. *„Ich muss in meinem Leben lernen, mit diesem Schmerz umzugehen“* (4, 00:16:02). *„Und immer wieder neu, sobald sich die Situation ändert“ *(4.1, 00:18:09). Dies ist geprägt von Zweifel und Hoffnungslosigkeit. *„Manchmal habe ich das Gefühl, das wird sich sowieso nicht ändern. Also die Schmerzen werden vielleicht noch schlimmer“* (5, 00:07:59). Die Frage ist entscheidend, wie chronischer Schmerz erklärt, aber auch gelindert und wie dessen beherrschende Rolle im Leben reduziert werden kann. *„Wo kommt der Schmerz her? Wieso bleiben die Schmerzen? Wieso gibt es so einen starken Schmerz?“* (4, 00:33:57). Im Umgang mit chronischem Schmerz entwickeln die Teilnehmenden unterschiedliche Strategien. Das kann Ablenkung sein, aber ebenso ein Fokussieren des Geistes. *„Meditieren, das hilft, gibt Entspannung. Und das ist für einen Schmerzpatienten wie eine Flucht, wo die Seele vor den Schmerzen flüchtet. Und es für eine kurze Zeit erträglich macht“* (2, 00:17:18). Neben Meditation werden auch Musik, Natur oder Gebet genannt. Dabei scheint nicht die Aktivität selbst ausschlaggebend zu sein, sondern dass die Aktivität zur eigenen Person passt. *„Es ist also entscheidend, dass jeder findet, was ihm hilft“* (3, 01:04:25).

Spiritualität stellt für viele Teilnehmende eine unverzichtbare Ressource im Umgang mit chronischem Schmerz dar, wie folgende Beispiele illustrieren. *„Also an den spirituellen Fragen komme ich gar nicht vorbei. Weil der Schmerz irgendwann die Existenz angräbt“* (2, 00:01:44). *„Meine Seele und mein Geist brauchen auch eine Dimension“* (5, 00:05:00). Die meisten Teilnehmenden setzen Spiritualität mit einer religiösen Überzeugung gleich. Die folgende Aussage steht exemplarisch für dieses Verständnis: *„Für mich gehört (Schmerz und Spiritualität) zusammen, weil ich weiß, ich kann einen Teil von meinen Schmerzen dem hinlegen, der mich geschaffen hat. Und das kann auch schon ein bisschen Schmerzen lösen“* (2, 00:08:11). Jemand anders sagte: *„Ich bete zum Beispiel jeden Tag. Für mich hat das etwas mit Spiritualität zu tun. Und ich bete jeden Abend, dass die Schmerzen weggehen. Also mit der Hoffnung, dass es eben eines Tages keine Schmerzen mehr gibt“* (5, 00:01:11). Diese Kopplung von Hoffnung auf Schmerzlinderung mit religiöser Überzeugung wurde auch kritisch gesehen. *„Wir haben die Schmerzen, und wir sind irgendwann vielleicht einmal erschöpft mit dem Glauben. Aber auf eine Art kannst du ja nicht sagen: Ich glaube jetzt nicht mehr, weil ich immer noch die Schmerzen habe“ *(5, 00:10:45). Manche Teilnehmende schilderten, dass das anhaltende Schmerzerleben ihren Glauben auf die Probe stellen kann*. „Je nachdem, wenn ich Schmerzen habe, denke ich: Ja, wo ist er jetzt und so? Und wenn ich dann (zur Therapie) muss und ich habe so *saumäßig *Schmerzen, wieso hilft er mir jetzt nicht?“ *(2, 00:46:10). Oder ihren Glauben gesamthaft infrage stellt: *„Gott hilft mir nicht. Er gibt mir keine Antwort“* (4.1, 00:33:57). Negative Aspekte oder Erfahrungen im Zusammenhang mit spirituellen bzw. religiösen Praktiken wurden nur vereinzelt zur Sprache gebracht. *„Also ja, es gibt viele Leute, zum Beispiel, die leiden unter der Religion. Ich bin auch Muslimin, aber ich leide gar nicht daran“ *(1, 00:14:44). *„Es gibt doch so die übersinnlichen Sachen da. Das zum Teil esoterische Zeug, wo doch einfach einen Menschen mehr zum Abgrund treibt“* (2, 00:26:09).

Spiritualität ausschließlich mit religiösen Überzeugungen gleichzusetzen, war für jene Teilnehmenden unpassend, die sich als nicht religiös bezeichneten*. „Ich habe einfach immer das Gefühl, die ganze Sache geht so sehr ein bisschen auf die religiöse Schiene, aber ich kann mich täuschen“* (5.1, 00:18:48). Vor allem Teilnehmende aus dieser Gruppe verbanden Spiritualität nicht mit religiöser Praxis, sondern mit alternativen Ressourcen, die ebenso Kraft spenden oder Sinn verleihen. *„Ich würde jetzt sagen, es ist nicht unbedingt Religion oder Gott oder so. Sondern es gibt einfach Kraft und vor allem die Natur zeigt mir das. Ich habe ein Erlebnis gehabt, wo ich so starke Schmerzen gehabt habe, dass ich gedacht habe, jetzt melde ich mich bei ‚Exit‘. Und ich bin aufgestanden, habe den Sonnenaufgang gesehen und gedacht: Nein! Das will ich weiter haben. Und auch die ganzen menschlichen Beziehungen, die ich habe“* (3, 00:04:23).

Die Teilnehmenden thematisieren noch einen weiterführenden Aspekt, der über die eigene Sinnfindung in der Schmerzerfahrung hinausgeht, nämlich ihre Erfahrungen und Erkenntnisse an andere weiterzugeben*. „Und dass wir nicht für unser Leben selber den Sinn finden, sondern dass wir den Sinn auch anderen geben können“ *(3, 00:08:15). Und das Teilen kann in positiver Weise auf die Betroffenen zurückkommen. *„Ich glaube, das hilft uns auch, wenn wir gebraucht werden in unserem Alltag. Unsere Erfahrungen haben sehr viel Wert und unsere Diagnose. Das ist etwas, das niemand anderes hat […] Das ist ein sehr großer Punkt. Und Du bist abgelenkt von deiner Welt“* (5, 01:04:08).

### „Ein Interaktionsraum mit Fachpersonen“ – Hinweise zum Einbezug der spirituellen Dimension in die Behandlung

Die Mehrheit der Teilnehmenden sprach sich dafür aus, der spirituellen Dimension in der Schmerzbehandlung einen gebührenden Platz einzuräumen. Die Teilnehmenden sehen ihre eigene Rolle im Behandlungsprozess durchaus kritisch. *„Manchmal sind wir sehr überfordert und werden immer die Schuld auf die Ärzte schieben und wir denken, die machen nichts […] Wir müssen wirklich versuchen, dass wir selber wenigstens etwas tun“* (1, 00:05:58). Aus ihrer Sicht liegt die Herausforderung für Fachpersonen jedoch genau darin, sich in die Lage des Menschen mit chronischen Schmerzen zu versetzen und auf dieser Grundlage empathisch und ganzheitlich zu handeln. Manchen Fachpersonen gelingt dies eher: *„Ich habe einen guten Arzt. Der schaut mich an und sagt: ‚Oh, oh, wir haben wieder Schmerzen‘“ *(3, 00:14:11). Anderen gelingt dies weniger oder gar nicht, wie eine Teilnehmerin ausführt: *„Und jeder (im Behandlungsteam) behandelt einfach nur den Punkt, wo er Fachmann ist. Er sieht nicht nach oben und nicht nach unten“ *(1, 00:25:52). Im Behandlungsprozess spiegelt sich die häufig erlebte Hilflosigkeit gegenüber der Person mit chronischen Schmerzen. *„Es ist ja vielmals die Hilflosigkeit. Die Hilflosigkeit von all den Menschen um mich herum […] Und ich denke, in der Behandlung ist das genau gleich“* (4.1, 00:23:36). Nicht nur einen ganzheitlichen, sondern auch einen interprofessionellen Ansatz vermissen Teilnehmende. *„Es muss einfach ein Team geben […] Und das Team muss einfach besser lernen miteinander zu arbeiten. Das muss rund werden“* (3, 00:48:55).

Von der Relevanz spiritueller Anliegen und Bedürfnisse in der Behandlung chronischer Schmerzen sind die meisten Teilnehmenden überzeugt. *„Ich finde das ganz wichtig, dass (die spirituelle Thematik) auch wirklich mindestens ein Drittel von der ganzen Betreuung einnehmen würde“* (4.1, 00:18:09). Sie betonen aber wiederholt, dass Patienten die Möglichkeit haben sollten, selbst darüber zu entscheiden, ob spirituelle Themen im Behandlungsprozess berücksichtigt werden sollen oder nicht. Sie sollten keinesfalls verordnet werden. *„(Die Fachpersonen) dürfen einem keine Sachen überstülpen, egal welche Religion. Ist die andere Person überhaupt spirituell oder nicht“ *(2, 00:52:07). Im Gegenteil, die patienteneigene Spiritualität soll hier ihren Platz einnehmen können: *„Einfach in dem, dass die Spiritualität (in der Behandlung) anerkannt wird, die man selber praktiziert, fühlt man sich wohler und die Schmerzen, auch die chronischen, werden weniger und man verkrampft sich weniger“ *(2, 00:17:28). Diesen Standpunkt teilen auch andere. *„Er (der behandelnde Arzt) muss ja nicht unbedingt davon überzeugt sein, dass es hilft oder so. Sondern er muss einfach davon überzeugt sein, dass er merkt, dass es uns hilft“* (3, 01:00:18). Genauso muss die Fachperson erkennen, ob der Patient der spirituellen Dimension in der Behandlung überhaupt Raum zugesteht: *„Man sollte das nicht generell einbeziehen. Weil das doch etwas sehr Persönliches ist“* (1, 00:02:04). Manche Teilnehmende ziehen es auch vor, Spiritualität in ihrem Behandlungsprozess auszuklammern, weil sie andernorts Unterstützung finden. *„Das habe ich gottlob in der Ehe. Also ich habe jetzt nicht das Bedürfnis, dass ich zu jemand anderen gehen muss“* (5.1, 00:03:53).

Authentizität in der Begegnung dient als Schlüssel, einen unmittelbaren Zugang zu den wesentlichen Themen zu öffnen, wie eine Teilnehmende ausführt: *„(Vor einem operativen Eingriff) bin ich zum Narkosearzt und also das ist unglaublich, der ist so offen gewesen, der hat dann sofort ein paar Fragen gestellt und gerade gewusst, wo ich stehe, wie es mir geht und eben auch mit dem Tod meiner Tochter. Und hat dann gesagt, er habe vor sieben Jahren seine Partnerin auch so plötzlich verloren, durch einen plötzlichen Tod. Und hat dann einfach von sich erzählt. Das ist unglaublich gewesen. Und dann hat er gesagt: ‚Schauen Sie, es ist wichtig, das kann ich Ihnen jetzt einfach aus Erfahrung sagen, dass wir jetzt einfach wieder aus dem Loch herauskommen und uns wieder zeigen im Dorf.‘ Das ist unglaublich gewesen, wie mir das geholfen hat. Und er hat gesagt, was ihm geholfen hat und hat eben auch Zeit gehabt“* (3, 00:58:49).

Grundsätzlich können sich die Teilnehmenden alle Fachpersonen aus allen Fachrichtungen als Ansprechpartner für spirituelle Themen im Rahmen ihres Behandlungsprozesses vorstellen. In ihren Beschreibungen der ambulanten Versorgung stehen die behandelnden Ärzte im Vordergrund. *„Ich habe einen sehr offenen Hausarzt, der sowieso auf spiritueller Ebene immer wieder anspricht. Vor allem, wenn ein Arzt weiß, dass man dem Patienten mit der Schulmedizin nicht mehr helfen kann“* (1, 00:21:43). In der stationären Rehabilitation oder in der häuslichen Umgebung fächern sich die Möglichkeiten breit auf. *„Das Pflegepersonal auf der Station. Das hat am meisten Kontakt“* (1, 00:53:01). *„Also eine Physiotherapeutin […] Dort kann ich nackt vor ihr stehen. Was ich bei der Ärztin nicht unbedingt gern mache“ *(5.1, 00:30:06, 00:31:23). *„Wenn man weiterverwiesen würde an einen Psychiater oder eine Psychologin für die Patienten, die noch nicht (weiterverwiesen) sind“* (2, 00:10:34). Aber nicht alle Fachpersonen verfügen zwingend über die entsprechenden Kompetenzen und Möglichkeiten, was Teilnehmende als normal verstehen. *„Ich fände es keine Schande, wenn ein Arzt mal sagt, mein Fachwissen hört da auf“* (1, 00:43:48). Auch in dieser Frage spielt Authentizität eine herausragende Rolle. *„Ich habe es erlebt, dass wenn einer an seine Grenzen kommt und sagt: ‚Ich weiß nicht mehr.‘ Die Ehrlichkeit ist eine Beziehung, die ich erlebe. […] Ich kann nur das geben, wo ich habe“ *(3, 00:47:27).

## Diskussion

Die Teilnehmenden gaben einen breiten Einblick in ihren Erfahrungsschatz als Person mit chronischen Schmerzen. Dies betrifft erstens ihre menschliche Existenz mit herausforderndem Symptomerleben („Der ganze Mensch“). Ein weiterer Punkt ist die Rolle spiritueller Ressourcen im Umgang mit den Symptomen, um ihnen einen angemessenen Platz im eigenen Leben zuzuweisen („Suchen und Wege finden“). Der dritte Aspekt ist der Wunsch, im Behandlungsprozess einen von Wertschätzung geprägten Raum zu haben, in dem auch spirituelle Themen zur Sprache kommen dürfen („Ein Interaktionsraum mit Fachpersonen“).

Zum Zeitpunkt der Interviews waren alle Teilnehmenden in einer auf chronischen Schmerz spezialisierten Praxis oder Einrichtung in Behandlung. Ihre Berichte, von Fachpersonen in ihrem Schmerzerleben nicht anerkannt zu werden, bezogen sich primär auf Erfahrungen in anderen Praxen oder Einrichtungen. Dies waren häufig Allgemeinpraxen, die sie, wie ein Großteil der Patientengruppe, bei Manifestation des chronischen Schmerzes aufsuchten [19]. Die geschilderten Erfahrungen schienen sich aber im Laufe der Zeit und im Zuge weiterer Abklärungen zu wiederholen. Dieser Sachverhalt erstaunt angesichts eines differenzierten und gut zugänglichen Gesundheitssystems und eines unter Fachpersonen allgemein bekannten und anerkannten Versorgungsmodells mit ganzheitlichem Anspruch, wie es das biopsychosoziale Modell darstellt [11], auch wenn dieses die spirituelle Dimension noch nicht mitberücksichtigt [31]. Das kann als Hinweis verstanden werden, dass sich dieses Modell, wie in anderen Ländern [6, 20, 31], auch in der Schweiz längst noch nicht flächendeckend durchgesetzt hat. Als ganzer Mensch in all seinen Dimensionen gesehen, anerkannt und behandelt zu werden, ist ein zentraler Aspekt in der therapeutischen Beziehung und nicht nur eine Beigabe [33].

Wie auch in anderen Studien beschrieben, berichteten die Teilnehmenden, dass chronischer Schmerz für sie real [20] und omnipräsent ist [6, 32]. Sozialer Rückzug und nachteilige wirtschaftliche Folgen durch Erwerbslosigkeit verschlimmern die Lage. Betroffenen mag es wie eine Zurückweisung erscheinen, wenn Fachpersonen ihr Schmerzerleben nicht anerkennen oder herunterspielen. Ebenso scheinen Fachpersonen genau wie Patienten in einen Sog zu geraten, fortgesetzt nach zugrunde liegenden pathophysiologischen Erklärungen für die Schmerzsymptomatik zu suchen, weil erst diese das Schmerzerleben zu legitimieren scheinen [14, 33].

Auch in unserer Stichprobe hat die plausible Erklärung für das Schmerzerleben für Betroffene eine entlastende Funktion. Doch greift eine ausschließlich biomedizinische Sicht auf das Leiden an chronischem Schmerz zu kurz [13, 34]. Zudem vermag die unaufhaltsame Suche nach pathophysiologischen Erklärungen und Lösungen darin behindern, sich auf Strategien einzulassen, mit chronischem Schmerz so gut wie möglich leben zu können [6]. Dies stellt jedoch einen grundlegenden Wendepunkt dar, der den heiklen Begriff „Akzeptanz“ mit sich führt. Akzeptanz als Kapitulation, als Aufgabe aller Hoffnung auf Besserung zu verstehen, liegt im Kontext chronischer Schmerzerkrankungen nahe [6]. Eine nichtresignative Form der Akzeptanz ist von der Erkenntnis geleitet, dass chronischer Schmerz zwar persistieren mag, er aber das Leben nicht beherrschen muss [29] und es Wege gibt, unausgeschöpfte Ressourcen zu erschließen, zu denen auch solche spiritueller Art gehören [1]. Der entscheidende Schritt könnte für Patienten und Fachpersonen sein, eine ergebnislose Suche nach pathophysiologischer Erklärung zu beenden oder zumindest durch eine neue Suchrichtung zu ergänzen [33]. Dies bedeutet keine Abkehr von allen biomedizinischen Möglichkeiten der Schmerzlinderung. Es bedeutet vielmehr eine Erweiterung der therapeutischen Möglichkeiten unter Einbezug der patienteneigenen spirituellen Ressourcen.

Eine vergleichbare Situation findet sich in der „palliative care“: Hier werden spirituelle Aspekte häufig in die Behandlung einbezogen, was aber nicht bedeutet, dass biomedizinische Therapien vernachlässigt werden. Wichtig ist, dass Patienten das Ziel dieses Übergangs verstehen und nach Möglichkeit mitgestalten können [15]. Auch in einer multimodalen Therapie chronischer Schmerzen sollten Fachpersonen und Patienten miteinander in einen offenen Dialog treten können, um gegenseitige Erwartungen zu klären, (auch spirituelle) Anliegen und Bedürfnisse auszudrücken, gemeinsame Behandlungsziele zu setzen und Strategien zur Zielerreichung zu entwickeln [19].

Die Teilnehmenden in unserer Untersuchung blieben vage, wie ihre spirituellen Bedürfnisse und Anliegen in Abgrenzung zur körperlichen, psychischen und sozialen Dimension konkret beschaffen sind. Dies entspricht allerdings analog berichteten Schwierigkeiten in der Forschungsliteratur und mag damit zu tun haben, dass Spiritualität individuell unterschiedlich gedeutet und mit dem chronischen Schmerz auf unterschiedliche Weise in Zusammenhang gebracht werden kann [9]. Unabhängig davon sagten sie, dass spirituelle Themen für den Behandlungsprozess hoch relevant sein können. Auffallend ist, dass in unserer Stichprobe 84 % (*n* = 35) einer religiösen Gemeinschaft angehörten und nur 16 % (*n* = 7) „konfessionslos“ angaben. Dies erklärt, weshalb sich die Beiträge zu spirituellen Bedürfnissen und Anliegen mehrheitlich auf religiöse Überzeugungen und Praktiken beziehen. Wie in der Studie von Gerbershagen et al. [12] bei neurologischen Schmerzpatienten scheint auch in unserer Stichprobe eine stark ausgeprägte Religiosität die Akzeptanz chronischer Schmerzen zu unterstützen. Inwieweit bei diesen Teilnehmenden auch negative religiöse Coping-Strategien zum Tragen kamen [2], geht aus den Interviewbeiträgen nicht hervor. Bemerkenswert ist, dass negative oder schädigende Aspekte und Erfahrungen unter den Teilnehmenden kaum zur Sprache kamen, während diese im Gegensatz dazu Gegenstand expliziter Diskussion unter den Fachpersonen waren [27]. Jedoch nahmen auch solche Beiträge Raum ein, die keinen Zusammenhang mit Religiosität herstellten. Der allgemeine Bezugspunkt waren hier Ressourcen der Hoffnung und der Kraft.

Die Teilnehmenden sprachen auch den Aspekt an, dass ihr Erleiden und ihr Umgang mit chronischem Schmerz einen Wert für Dritte darstellen kann. Denn ihre Erfahrungen vermitteln einen unmittelbaren Einblick in ein Leben mit chronischem Schmerz aus der Perspektive direkt Betroffener [14], der sowohl Fachpersonen als auch anderen Betroffenen zugutekommen kann. Gleichwohl kommt hier eine Verbundenheit mit anderen bzw. Transzendenz zum Ausdruck, die einen wesentlichen Bestandteil der spirituellen Dimension darstellt [2, 12].

## Limitationen

Personen mit akut bedrohlicher Erkrankung, wozu wir auch Krebserkrankungen zählten, waren von einer Teilnahme ausgeschlossen. Wir wähnten unter solchen Umständen die Thematik des Lebensendes im Vordergrund, was den Blick auf Spiritualität im Zusammenhang mit chronischen Schmerzen verdecken kann. Damit blieben in der Rekrutierung auch Patienten mit einer langjährigen, stabilen Krebsdiagnose unberücksichtigt, bei denen die Thematik des Lebensendes möglicherweise in den Hintergrund getreten ist.

Die Mehrzahl der Teilnehmenden gab die Zugehörigkeit zu einer Religionsgemeinschaft an, wobei offenblieb, inwieweit dies ihre Beiträge zu spirituellen Bedürfnissen und Anliegen prägte. Dennoch könnten dadurch in den Ergebnissen religiöse Einstellungen überrepräsentiert sein. Die Offenheit in der Moderation ließ zu, dass Beiträge Raum einnahmen, die über das eigentliche Forschungsthema spiritueller Bedürfnisse und Anliegen hinausgingen. Einerseits konnte dadurch ein breiteres Bild der Gesamterfahrung in dieser Patientengruppe skizziert werden, andererseits ging dies möglicherweise auf Kosten einer detaillierteren Darstellung spiritueller Themen. Schließlich könnten die fokussierten Leitfragen die Teilnehmenden veranlasst haben, sich überwiegend positiv bezüglich der Integration spiritueller Anliegen und Bedürfnisse auszudrücken, sodass kritische Einstellungen oder Formen eines negativen religiösen Copings eher im Hintergrund blieben. Dies ist in der Interpretation der Ergebnisse zu berücksichtigen.

## Fazit für die Praxis

Grundbausteine in der Interaktion zwischen Fachpersonen und Patienten mit chronischen Schmerzen sind:

- Den ganzen Menschen auch in seiner spirituellen Dimension anzuerkennen und den bisherigen und aktuellen Umgang mit dem Schmerzerleben zu würdigen
- Im Rahmen einer multimodalen Schmerztherapie auch spirituelle Aspekte zu berücksichtigen
- Patienten auf dem Weg zu einer nichtresignativen Akzeptanz des Schmerzerlebens unter Einbezug auch spiritueller Ressourcen zu begleiten

## Footnotes

1 Der Code bezeichnet das Studienzentrum und den Zeitpunkt der Aussage im Interview.
